# Supplementary material for: COZOID: contact zone identifier for visual analysis of protein-protein interactions
Source: BMC Bioinformatics. 2018 Apr 6;19:125. doi: 10.1186/s12859-018-2113-6 (PMC5889581; doi:10.1186/s12859-018-2113-6)
Supplement: Supplementary file 2 — Software build. Executable binary file of the software tool. (ZIP 118,784 kb) [file 12859_2018_2113_MOESM2_ESM.zip › cozoid/java/docs/beansbinding-1.2.1-doc.zip!/org/jdesktop/swingbinding/impl/ListBindingManager.html]

ListBindingManager (Beans Binding)


---


|  |  |  |
| --- | --- | --- |
| **Overview** \| **Package** \| **Class** \| **Use** \| **Tree** \| **Deprecated** \| **Index** \| **Help** | |  |
| **PREV CLASS**   NEXT CLASS | **FRAMES**    **NO FRAMES**     **All Classes** |  |
| SUMMARY: NESTED \| FIELD \| CONSTR \| METHOD | DETAIL: FIELD \| CONSTR \| METHOD |  |


---


## org.jdesktop.swingbinding.impl Class ListBindingManager

```
java.lang.Object
  org.jdesktop.swingbinding.impl.ListBindingManager
```

**All Implemented Interfaces:**: java.util.EventListener, ObservableListListener

---

``` public abstract class ListBindingManager extends java.lang.Object implements ObservableListListener ```

---

**Constructor Summary**

`ListBindingManager()`


| **Method Summary** | |
| --- | --- |
| `protected abstract  void` | `added(int index, int length)` |
| `protected abstract  void` | `allChanged()` |
| `protected abstract  void` | `changed(int row)` |
| `int` | `columnCount()` |
| `protected abstract  AbstractColumnBinding[]` | `getColBindings()` |
| `java.lang.Object` | `getElement(int index)` |
| `java.util.List<?>` | `getElements()` |
| `void` | `listElementPropertyChanged(ObservableList list, int index)`             Notification than a property of an element in this list has changed. |
| `void` | `listElementReplaced(ObservableList list, int index, java.lang.Object oldElement)`             Notification that an element has been replaced by another in the list. |
| `void` | `listElementsAdded(ObservableList list, int index, int length)`             Notification that elements have been added to the list. |
| `void` | `listElementsRemoved(ObservableList list, int index, java.util.List elements)`             Notification that elements have been removed from the list. |
| `protected abstract  void` | `removed(int index, int length)` |
| `void` | `setElements(java.util.List<?> elements, boolean sendAllChanged)` |
| `int` | `size()` |
| `java.lang.Object` | `valueAt(int row, int column)` |
| `protected abstract  void` | `valueChanged(int row, int column)` |

**Methods inherited from class java.lang.Object**

`clone, equals, finalize, getClass, hashCode, notify, notifyAll, toString, wait, wait, wait`

**Constructor Detail**

### ListBindingManager

```
public ListBindingManager()
```


**Method Detail**

### getColBindings

```
protected abstract AbstractColumnBinding[] getColBindings()
```

---


### setElements

```
public void setElements(java.util.List<?> elements,
                        boolean sendAllChanged)
```

---


### getElement

```
public final java.lang.Object getElement(int index)
```

---


### getElements

```
public final java.util.List<?> getElements()
```

---


### size

```
public final int size()
```

---


### valueAt

```
public final java.lang.Object valueAt(int row,
                                      int column)
```

---


### columnCount

```
public final int columnCount()
```

---


### listElementsAdded

```
public final void listElementsAdded(ObservableList list,
                                    int index,
                                    int length)
```

:   **Description copied from interface: `ObservableListListener`**
:   Notification that elements have been added to the list.

    :   **Specified by:**: `listElementsAdded` in interface `ObservableListListener`
    :   **Parameters:**: `list` - the `ObservableList` that has changed: `index` - the index the elements were added to: `length` - the number of elements that were added

---


### listElementsRemoved

```
public final void listElementsRemoved(ObservableList list,
                                      int index,
                                      java.util.List elements)
```

:   **Description copied from interface: `ObservableListListener`**
:   Notification that elements have been removed from the list.

    :   **Specified by:**: `listElementsRemoved` in interface `ObservableListListener`
    :   **Parameters:**: `list` - the `ObservableList` that has changed: `index` - the starting index the elements were removed from: `elements` - a list containing the elements that were removed.

---


### listElementReplaced

```
public final void listElementReplaced(ObservableList list,
                                      int index,
                                      java.lang.Object oldElement)
```

:   **Description copied from interface: `ObservableListListener`**
:   Notification that an element has been replaced by another in the list.

    :   **Specified by:**: `listElementReplaced` in interface `ObservableListListener`
    :   **Parameters:**: `list` - the `ObservableList` that has changed: `index` - the index of the element that was replaced: `oldElement` - the element at the index before the change

---


### listElementPropertyChanged

```
public final void listElementPropertyChanged(ObservableList list,
                                             int index)
```

:   **Description copied from interface: `ObservableListListener`**
:   Notification than a property of an element in this list has changed.
    Not all `ObservableLists` support this notification. Only
    observable lists that return `true` from
    `supportsElementPropertyChanged` send this notification.

    :   **Specified by:**: `listElementPropertyChanged` in interface `ObservableListListener`
    :   **Parameters:**: `list` - the `ObservableList` that has changed: `index` - the index of the element that changed

---


### allChanged

```
protected abstract void allChanged()
```

---


### valueChanged

```
protected abstract void valueChanged(int row,
                                     int column)
```

---


### added

```
protected abstract void added(int index,
                              int length)
```

---


### removed

```
protected abstract void removed(int index,
                                int length)
```

---


### changed

```
protected abstract void changed(int row)
```


---


|  |  |  |
| --- | --- | --- |
| **Overview** \| **Package** \| **Class** \| **Use** \| **Tree** \| **Deprecated** \| **Index** \| **Help** | |  |
| **PREV CLASS**   NEXT CLASS | **FRAMES**    **NO FRAMES**     **All Classes** |  |
| SUMMARY: NESTED \| FIELD \| CONSTR \| METHOD | DETAIL: FIELD \| CONSTR \| METHOD |  |


---
